# A Supportive-Educational Intervention for Heart Failure Patients in Iran: The Effect on Self-Care Behaviours

**DOI:** 10.1155/2013/492729

**Published:** 2013-09-22

**Authors:** Vahid Zamanzadeh, Leila Valizadeh, A. Fuchsia Howard, Fatemeh Jamshidi

**Affiliations:** ^1^Department of Medical-Surgical Nursing, Faculty of Nursing and Midwifery, Tabriz University of Medical Sciences, Tabriz, Iran; ^2^Department of Pediatric Nursing, Faculty of Nursing and Midwifery, Tabriz University of Medical Sciences, Tabriz, Iran; ^3^School of Population and Public Health, Faculty of Medicine, University of British Columbia, Vancouver, Canada; ^4^Cardiovascular Research Center, Tabriz University of Medical Sciences, Tabriz, Iran

## Abstract

*Background*. Chronic heart failure is a major health and social problem. The promotion of self-care behaviours can potentially assist patients to effectively manage this chronic condition and prevent worsening of the disease. Formal personalized educational interventions that provide support and take into consideration the cultural context are needed. *Objective*. The objective of this research was to evaluate the effect of a supportive-educational intervention on self-care behaviours of heart failure patients in Iran. *Methods*. This research was a prospective, randomized trial of a supportive-educational intervention. Eighty heart failure patients were randomly assigned to receive the supportive-educational intervention or usual care. The intervention consisted of a one-hour, nurse-led, in-person education session and postdischarge followup by telephone over three months. Data were collected at baseline, one, two, and three months. *Results*. The control and intervention groups did not differ in self-care scores at baseline (*P* > 0.05). Each of the self-care scores was significantly higher in the intervention group than the control group at 1, 2, and 3 months (*P* < 0.001). There were significant differences in self-care behaviours over the three months, among participants in the intervention group. *Conclusion*. This study provides support for the effectiveness of a supportive-educational intervention to increase self-care behaviours among Iranian patients suffering from chronic heart failure.

## 1. Introduction

Chronic heart failure (HF) is a significant health and social problem [1]. In the United States alone, nearly 6 million people suffered from HF in 2008, and this disease is becoming increasingly prevalent [2] with more than 550,000 new cases diagnosed each year [3]. Half of HF patients die within 5 years of the first onset of symptoms, and half (50%–60%) of the patients diagnosed with severe HF do not live longer than a year [4]. HF is the most common cause of hospitalization in those over the age of 65 years [5], and 54% of the patients are readmitted to hospital within 6 months of discharge [6]. HF also results in significant morbidity and disability, thereby generating permanent and high health care costs [7]. Heart disease is the leading cause of mortality in Iran [8]; unfortunately, further accurate statistics describing the burden of HF are not available.

Optimal medical management following a cardiovascular event remains underprescribed, and even more so in developing countries [8]. Similarly, nonpharmacological management and interventions are infrequently recommended, and patient adherence to lifestyle modifications remains poor. Recent European research suggests that the control of cardiovascular risk factors, including tobacco use, obesity, hypertension, hypercholesterolemia, and diabetes, is far below an acceptable level [9, 10].

The nature and severity of HF symptoms greatly depend on the patient's knowledge, cooperation, and active participation in their health management. However, improving health and preventing HF from progressing by adopting self-care skills, adhering to complex treatment regimens, and changing lifestyle behaviours is particularly challenging [3].

According to orem's self-Care theory, effective self-care activities can reduce the need for hospitalization [11]. This theory postulates that nurses interact with patients in three ways according to the patient's ability to participate in their care: total compensation, partial compensation, and supportive-educational systems. Patients in the supportive-educational system are capable of engaging in self-care but require education about the different aspects of therapeutic self-care behaviours [12]. Nurses are optimally positioned to identify existing and potential health issues and to provide supportive-educational interventions where appropriate [13].

Postdischarge support in the form of patient education is one of the most effective interventions to improve self-care abilities and behaviours among HF patients, which ultimately improves prognosis and reduces hospital readmission rates [13]. The main objective of education for HF patients is to improve the patient's management of their disease, thereby reducing the onset of complications and morbidity [14]. Patient education is generally delivered within the framework of a comprehensive discharge program and covers information about fluid and sodium intake restriction, diet, exercise, adherence to pharmaceutical treatment, monitoring symptoms, and seeking health care when symptoms worsen [15]. According to a recent systematic review [14], large studies have demonstrated the efficacy of therapeutic education programs in changing cardiac patients' lifestyles and ultimately improving morbidity and cost-effectiveness. Yet, there are no recommendations or standardized guidelines about methods to deliver information and education.

Strömberg [5] found that most HF patients do not have a clear understanding of recommended self-care behaviours despite receiving related education. Rather, HF patients require further assistance to learn self-care behaviours and adapt to living with a chronic illness. Providing education and training based on individual patient needs and desires is an essential principle in adult education. Therefore, a thorough assessment of HF patients' educational needs and preferences, as well as their beliefs and abilities related to medical and lifestyle recommendations, can provide the foundation for personalizing educational efforts [16]. 

Interventions to promote self-care behaviours among HF patients, and corresponding research, must take into account culture, which greatly influences diet, exercise, lifestyle, and attitudes toward medical therapy [13]. The few intervention studies conducted in Iran did not evaluate supportive-educational interventions nor did they measure self-care behaviours [17–19]. The purpose of this research was to evaluate the effect of a supportive-educational intervention on self-care behaviours of Iranian HF patients.

## 2. Methods

### 2.1. Design and Setting

The Research Council of the Tabriz University of Medical Sciences gave ethical approval for this randomized control trial. Written informed consent was obtained for all participants prior to study enrollment. All participants were recruited from the Shahid Madani Hospital, located in Eastern Iran.

### 2.2. Sample and Randomization

**Figure 1:**
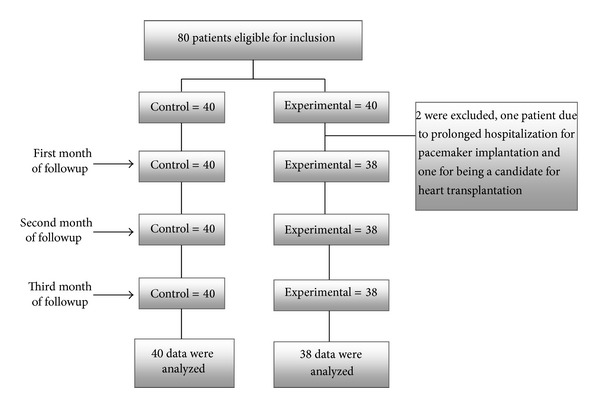
Randomization flow chart.

Consecutive patients admitted to Shahid Madani Hospital with a diagnosis of HF and who met the inclusion criteria were recruited into the study. A sample size of 80 (40 individuals in the intervention group and 40 in the control group) was deemed sufficient based on a preliminary analysis of self-care scores of 5 HF patients. The following parameters guided the present study; the optimal self-care behaviour score in the study was 70, the mean and standard deviation of self-care behaviours scores were estimated (Mean = 25, SD = 6.15), *α* = 0.05 and power = 0.9 were chosen, and no attrition during followup was anticipated. The participants were randomized into the control and experimental groups using random number software (Figure 1).

### 2.3. Inclusion Criteria

Participants who were included were of 18 years age and older, diagnosed with New York Heart Association class III or IV HF, had an ejection fraction less than 40%, agreed to predischarge education and follow-up care, and would be available by phone after discharge.

### 2.4. Exclusion Criteria

Participants who were excluded were those who experienced significant worsening of their disease and transfer to the intensive care unit, were hospitalized for greater than 1 month, had a chronic disease other than HF, or were diagnosed with a mental illness.

### 2.5. Study Intervention

According to orem's self-care theory, the supportive-educational system is the only system in which patients require assistance in relation to decision-making, behaviour control, and acquiring knowledge and skills. However, the level and type of assistance/care required can vary. Some patients are capable of carrying out self-care behaviours but are in need of guidance and support, while others only require education. Still, others adequately engage in self-care and only require periodic guidance [12]. The supportive-educational intervention in the present study was tailored to address the appropriate level and type of assistance/care as per the participant's need. Those who needed guidance and support were encouraged to continue to carry out current self-care behaviours, and additional information and support were provided related to reducing salt in the diet, restricting fluids, and increasing physical activity for example. Participants requiring education were given information about how to create new self-care behaviours. Participants only requiring periodic guidance were given frequent tips and advice via telephone. 

HF participants randomized to the intervention group received a two-part intervention aimed at improving self-care behaviours. The first phase consisted of a one-hour, nurse-led, in-person HF education session that was customized by the nurse according to the participant's level of education. An individualized education booklet was reviewed with literate patients, while for illiterate patients this booklet was reviewed with the participant as well as a family member. The intervention was also customized according to the participant's prior knowledge and learning needs, which was assessed with a learning need inventory. Participants and their family members attended this session. During the education session the following information was reviewed: the definition and symptoms of HF, strategies to prevent the worsening of HF symptoms, explanations about medications, and recommendations about dietary changes (i.e., reducing salt intake), exercise, and smoking cessation. These participants were given a booklet at the time of discharge that was based on Heart Failure Society of America (HFSA) 2010 Guideline Executive Summary.

The second phase of the intervention included postdischarge telephone followup. The objective of this phase was to reiterate and review information covered during the initial education session and improve the participant's ability to cope with the disease, as well as enhance self-care behaviours. The first followup telephone call was made by a nurse two days after hospital discharge to verify participant information and determine the next date of contact. The nurse then contacted the participant by phone every two weeks for 3 months. During these phone calls the nurse asked the participant whether they were experiencing any signs or symptoms that would suggest worsening HF. The nurse also reviewed the recommended self-care behaviours and provided support in the form of advice and encouragement when deemed necessary. These follow-up telephone calls typically lasted 15 minutes. The participants were also advised to contact the nurse if any question or an acute medical issue arose related to preventing or managing their HF.

Participants who were randomized to the control group received usual care provided by the hospital and attending physician (nonsystematic and informal teaching).

### 2.6. Assessment Tools

Sociodemographic characteristics were gathered through individual interviews and medical data were extracted from medical records. 

The educational needs of participants in the intervention group were assessed with a self-report questionnaire, which was filled out independently with pen and paper or with the assistance of a nurse during a face-to-face interview. The education needs assessment instrument [20] is specific to HF patients and consists of 42 items, with 7 subscales to assess learning needs in the areas of anatomy, physiology, diet, activity, cognitive factors, risk factors, and pharmaceutical information. This instrument uses a five-point Likert scale (ranging from least important to know = 1 to most important to know = 5). 

Baseline self-care behaviour data were collected with the pen and paper method or during face-to-face interviews using the self-care of heart failure index (SCHFI) [21]. Subsequent self-care behaviour data were collected with this instrument by telephone at 1, 2, and 3 months following hospital discharge. Completion of the SCHFI took 15 minutes. The SCHFI consists of 22 items in three subscales. The maintenance self-care behaviours scores includes 10 questions, the self-care management the scores includes six items, and the self-care confidence the scores includes seven questions. All items are scored on a four-point Likert scale from 1 (poor self-care behaviour) to 4 (optimal self-care behaviour), and summative scores are standardized on a scale of 0 to 100. A cutoff point of ≥70 on each SCHFI scale is used to judge self-care adequacy [21].

To ensure the accuracy of the Farsi translation of these instruments, they were reviewed by three professors (two with English language M.A. degrees and one with an M.S. degree in nursing) and revised accordingly. The instruments were reviewed for content validity by 12 faculty members of Tabriz University of Medical Sciences (9 with an M.S. and 2 with a Ph.D. in nursing, one of whom specializes in cardiovascular care). The reliability was determined through test-retest methods, wherein the instruments were given to 10 HF patients twice, 2 days apart. The correlation coefficient between these two time points was 96%. These patients were not participants in the current study.

### 2.7. Statistical Analysis

Measures of central tendency mean, standard deviation, and percentage were used to describe participant characteristics at baseline. The control and intervention groups' self-care behaviours were compared at baseline, 1, 2, and 3 months using *t*-tests. A repeated-measures ANOVA was used to determine time effects and the impact of group on different aspects of self-care behaviours. The significance level was set at *P* < 0.05.

## 3. Results

### 3.1. Sample Characteristics

A total of 200 HF patients were screened for inclusion from July to September 2011. 80 HF patients with an ejection fraction above 40% were excluded, 20 patients with severe HF were transferred to another hospital unit, and 20 patients declined to participate. The 80 remaining patients were enrolled in the study and randomized into the control (*n* = 40) or intervention (*n* = 40) groups. Of the 40 individuals assigned to the intervention group, two did not complete the study; one participant required pacemaker implantation, and another became a heart transplant candidate. At study completion, 38 individuals remained in the intervention group and 40 in the control group. 

The mean age of participants was 63.5 years. 53.8% were men, and 72.5% were married and lived with their spouse and children. Ischaemic heart disease (36%) and hypertension (36%) were the main causes of participant's HF. Participant demographic characteristics are summarized in Table 1. There was no statistically significant difference between sociodemographic characteristics of the participants in the control and intervention groups.

### 3.2. Self-Care Behaviours

The control and intervention groups did not differ in self-care scores at baseline, prior to delivery of the intervention. Each of the self-care scores was significantly higher in the intervention group than the control group at 1, 2, and 3 months (Table 2). The ANOVA results showed significant differences in self-care between the control and intervention groups (Tables 3 and 5). The results also showed significant difference in self-care behaviours over the three months, such that as time progressed self-care scores among participants in the intervention group continued to increase (Table 4).

## 4. Discussion

This study provides evidence that a supportive-educational intervention can strengthen and establish new self-care behaviours among HF patients in Iran. This intervention, informed by Orem's self-care theory, provided patient education about self-care behaviours to assist in the management of HF, as well as support in the form of telephone followups. This improvement in self-care behaviours is consistent with previous research, wherein self-care skills among HF patients were improved following one educational session and an eight-month followup with a nurse educator [1]. Additional advantages of educational interventions have been documented. Strömberg and colleagues [4] found that the readmission rate and healthcare costs also decreased with educational interventions that included follow-up support for HF patients. This provides support for including education about self-care activities, including nonpharmaceutical interventions, as a part of standard management of hospitalized HF patients. This educational training ought to begin when patients are initially hospitalized and continue following discharge.

In the present study, improvements in self-care among participants in the intervention group were not only maintained, but continued to improve over the three months. This finding lends support to comments made by Evangelista [3] that education alone does not lead to positive outcomes, and that using behavioural strategies, such as reinforcing behaviours through a follow-up program, can help optimize self-care. However, it is unknown how long beyond the 3-month followup the improvements made in self-care in the present study lasted among participants in the intervention group. There is evidence that the effect of educational interventions on self-care behaviours is not maintained over time [22–24]. 

## 5. Limitations and Suggestions

It was likely that the participants gave incorrect answers to the self-report questions. However, by gaining their trust and explaining the confidential nature of the study, we attempted to control false reporting. Patient education is currently an essential part of treating chronic diseases such as HF, but, in the busy day-to-day practice of caring for HF patients, education often is not a priority. We recommend that time and resources are to be allocated to enable health care professionals to adequately promote patients' self-care behaviours through supportive-educational interventions. The present research also suggests that further knowledge about factors that limit or promote effective patient education is needed to improve their quality and effectiveness.

## 6. Conclusion

Findings of this study indicated that patients with HF not only need pharmaceutical management by physicians and nurses, but they also require support to enhance their self-care behaviours and non-pharmaceutical management (e.g., reducing salt in the diet, restricting fluid intake, daily weighing, and monitoring the symptoms). According to the results of the present study, implementing personalized, theoretically driven, supportive-educational programs based on nonpharmacological management strategies might be a useful tool to develop, maintain, and change self-care behaviours of HF patients. This study confirmed that postdischarge supports are very effective in improving self-care activities and reaching optimal behaviours.

## Figures and Tables

**Table 1:** Patient demographics.

	Intervention group (*n* = 38) Mean ± SD or *n* (%)	Control group (*n* = 40) Mean ± SD or *n* (%)	Statistics
Age in years	65.82 ± 9.87	61.63 ± 12.47	*t* = 1.63
			df = 76
			*P* = 0.10
Gender			*x* ^2^ = 1.63
Male	24 (57.9)	19 (47.5)	df = 1
Female	16 (42.1)	21 (52.5)	*P* = 0.38
Educational level			
Illiterate	20 (52.6)	26 (65)	*x* ^2^ = 6.44
Primary	12 (31.6)	5 (12.5)	df = 5
High school	6 (19.4)	6 (15)	*P* = 0.26
University	0	1 (2.5)	
Marital status			
Single	0	2 (5)	*X* ^2^ = 4.19
Married	31 (81.6)	35 (87.5)	df = 3
Widowed, divorced	7 (18.2)	3 (7.5)	*P* = 0.24
Occupation			
Housewife	16 (42.1)	21 (52.5)	*X* ^2^ = 2.61
Employee	1 (2.6)	2 (5)	df = 3
Private	12 (31.6)	7 (17.5)	*P* = 0.24
Unemployed, and so forth	9 (22.7)	10 (25)	

Heart failure illness and treatment characteristics			

NYHA functional class			*x* ^2^ = 0.05
III	20 (52.6)	18 (45)	df = 1
IV	18 (47.4)	22 (55)	*P* = 0.82
Ejection fraction	25.73 ± 9.20	24.05 ± 8.94	*t* = 0.5
			df = 75
			*P* = 0.61
Aetiology			
Ischaemic	11 (28.9)	15 (37.5)	*x* ^2^ = 5.01
Hypertensive & dilated	18 (47.4)	12 (20)	df = 6
Cardiomyopathy & valvular	8 (21)	8 (20)	*P* = 0.54
Medication			
Diuretic	6 (15)	4 (10)	*X* ^2^ = 0.92
Beta blockers and diuretic	11 (28.9)	9 (22.5)	df = 2
Digoxin and diuretic	21 (55.3)	27 (67.5)	*P* = 0.63

**Table 2:** Comparison of self-care scores (maintenance, management, and confidence) by group and time.

	Intervention Mean ± SD	Control Mean ± SD	Student's *t*-test, *P* value
Self-care maintenance			
Baseline	18.5 (12)	21.9 (14.6)	−1, >0.05
1st month	56.6 (25)	23.8 (15)	6.9, <0.001
2nd month	70.2 (21.3)	30.5 (16.4)	9.2, <0.001
3rd month	75.1 (20.7)	31.9 (15.5)	10.4, <0.001
Self-care management			
Baseline	11.9 (11.9)	16.7 (16.7)	−1.4, >0.05
1st month	48.9 (20.5)	21.5 (16.7)	6.4, <0.001
2nd month	61.1 (18.5)	28.2 (17.4)	8, <0.001
3rd month	66.5 (15.3)	30.3 (17.6)	9.6, <0.001
Self-care confidence			
Baseline	10.6 (13.3)	16.8 (14.4)	−1.9, >0.05
1st month	53.5 (24.6)	18.3 (16.5)	7.3, <0.001
2nd month	66.1 (23.2)	23.6 (17)	9.1, <0.001
3rd month	69.6 (25.3)	27.6 (18.6)	8.3, <0.001

**Table 3:** ANOVA test for comparisons of changes in self-care before the intervention and during the first, second, and third months after the intervention in the intervention and control groups.

Change source	Type III sum of squares	df	Mean square	*F*	Sig.
Month effect	54786.96	2.13	25687.18	228.02	<0.001
Group effect	54610.00	1	54610.00	61.166	<0.001
Within-subjects contrasts	26148.27	2.13	12259.77	108.82	<0.001
Within-subjects errors	18260.55	162.09	112.65	228.02	
Between-subjects errors	67854.36	76	892.82	61.16	

**Table 4:** Bonferroni post hoc test for pairwise comparisons of changes in self-care before the intervention and during the first, second, and third months after intervention.

(I) Month	(J) Month	Mean difference (I-J)	Std. error	Sig.
0	**1**	−21 (*)	1.59	<0.001
	**2**	−30.53 (*)	1.72	<0.001
	**3**	−34.10 (*)	1.78	<0.001
1	**2**	−9.53 (*)	0.97	<0.001
	**3**	−13.09 (*)	1.30	<0.001
2	**3**	−3.56 (*)	0.98	0.003

*The mean difference is significant at the 0.05 level.

**Table 5:** Bonferroni post hoc test for comparison of two groups for self-care change.

(I) Group	(J) Group	Mean difference (I-J)	Std. error	Sig.
Intervention	Control	26.46 (*)	3.28	<0.001
